# Shall the Medical Association of Georgia Be Located?

**Published:** 1885-06

**Authors:** 


### Editorial.

### SHALL THE MEDICAL ASSOCIATION OF GEORGIA BE LOCATED?

The State Medical Association, at its late meeting in Savannah, raised a committee to consider the propriety of fixing the meetings of the Society, in future, at some one point, and to recommend a place for its permanent home. This is an important question, and without forestalling the action of that committee, it would seem proper that there should be some interchange of thought by the members living in different parts of the State.

The advantages of a permanent place of meeting are obvious in the better preservation of its archives, the more prompt and better editing and publication of its papers, and possibly the more regular conduct and dignity of its proceedings. But, on the other hand, the idea which originally determined the migratory feature of its meeting still has force. It was the hope of interesting in its deliberation a larger number of the profession and securing the presence, at least occasionally, of a greater number of members at its annual meetings. This result has been partially accomplished, as is shown by the list of admissions made at various and distant towns in which the Association has assembled.

It is believed that the Association is about entering upon an era of greater popularity and a wider field of usefulness, and that it may be so conducted and modified that it will arouse the pride and enlist the earnest co-operation of every physician in the State.

Would not the advantages of a permanent location be sufficiently secured by having every alternate meeting held at a fixed point, and the others, as at present, in such localities as the Association may determine?

The expensive hospitalities which custom, it is thought, demands upon these occasions are keenly enjoyed and gratefully remembered by the visiting brethren, but they are somewhat onerous to the profession and to the generous population of small towns, in which it might be desirable to hold an annual meeting. This difficulty should be obviated by abolishing the custom, and announcing that, at least, at the peripatetic meetings, such entertainments were not expected nor desired.

